# Computational corrections for anisotropic absorption in tensor tomography

**DOI:** 10.1107/S1600577525008641

**Published:** 2025-10-28

**Authors:** Mads Carlsen, Marianne Liebi

**Affiliations:** a Center for Photon Science Paul Scherrer Institut 5232Villigen PSI Switzerland; b Institute of Materials École Polytechnique Fédérale de Lausanne (EPFL) 1015Lausanne Switzerland; University of Malaga, Spain

**Keywords:** X-ray diffraction computed tomography, tensor tomography, anisotropic absorption, X-ray scattering

## Abstract

In X-ray scattering tensor tomography at large scattering angles, the absorption of scattered X-rays by the sample itself is anisotropic due to the macroscopic geometry of the sample. This effect is mostly ignored or only treated approximately in established reconstruction algorithms. In this paper we perform a simulation study to estimate the severity of the problem and suggest and test a computational approach to correct for this effect. We also investigate experimental scattering data from hydroxyapatite scattering from a piece of beaver tooth where the same trends from the simulations can be observed. We conclude that the conventional approach to transmission correction yields good results at scattering angles and levels of absorption normally used.

## Abstract 2

We investigate the effect of anisotropic absorption of scattered X-rays in X-ray diffraction tomography and show that the detrimental effect on reconstruction is small in the range of absorption coefficients and scattering angles commonly used.

## 1. Introduction

In traditional X-ray computed tomography (CT), the absorption density of the sample is the quantity being measured. In contrast to this, X-ray diffraction computed tomography (XRD-CT) (Kleuker *et al.*, 1998 ▸) and related synchrotron X-ray techniques such as small-angle X-ray scattering computed tomography (SAXS-CT) (Schroer *et al.*, 2006 ▸) measure the scattering density of the sample, and the absorption of X-rays by the sample is a source of error that has to be corrected for in the reconstruction if the absorption is large.

In these techniques, the measured intensity is modeled by a sum of contributions from individual voxels along the path of the direct X-ray beam. However, both the incident and scattered X-rays are absorbed by the sample before they can be measured. The path length of the direct beam through the sample inevitably varies during the experiment as the sample is translated and rotated. Furthermore, when X-rays are scattered to large angles, rays scattered in different directions have different path lengths and therefore the scattered X-ray signal is absorbed anisotropically.

This issue was discussed by Kleuker *et al.* (1998 ▸) in the setting of XRD-CT. The authors used a computational approach to correct for the absorption where the absorption is computed for each voxel and a range of different scattering directions. Such a computational correction is bothersome for several reasons, and in XRD-CT today absorption effects are usually either ignored, when absorption is low, or a simpler correction scheme is utilized, when absorption is significant, where the path of the scattered X-rays is assumed not to deviate too much from the path of the transmitted ray and the measured scattering can be normalized by the transmission of the direct beam. This correction is by some authors referred to as the zero-order approximation (Vamvakeros *et al.*, 2021 ▸).

In X-ray scattering tensor tomography (TT) (Liebi *et al.*, 2015 ▸; Schaff *et al.*, 2015 ▸) the issue of anisotropic absorption is thought to be more severe, as the anisotropy of scattered X-rays is the fundamental quantity of interest and anisotropy caused by absorption is therefore liable to be misinterpreted as anisotropic scattering properties of the sample material. In small-angle X-ray scattering tensor tomography (SASTT), the small-angle approximation is justified as it can be assumed that the path of the scattered ray differs little from the path of the direct beam when the scattering angle 2θ is small. In wide-angle X-ray scattering tensor tomography (WASTT) (Grünewald *et al.*, 2020 ▸), however, this approximation cannot so easily be justified. Grünewald *et al.* (2023 ▸) have discussed the issue of anisotropic absorption and describe a computational absorption correction where all scattering occurring along the direct beam is assumed to see the same absorption as for a voxel at the central point along the beam path, thereby allowing the absorption correction to be done as a pre-processing step before the TT reconstruction.

While we investigate the anisotropic absorption problem in the setting of WASTT, similar concerns are present in other X-ray scattering tomography techniques that operate at large scattering angles and use the intensity of the scattered X-ray. These include certain versions of scanning 3D XRD (Bonnin *et al.*, 2014 ▸; Hektor *et al.*, 2019 ▸), texture tomography (Frewein *et al.*, 2024 ▸) and strain–stress TT (Modregger *et al.*, 2025 ▸).

In X-ray fluorescence tomography, the absorption issue has received more attention than in scattering based methods since the photon energy of the fluoresced X-rays is fixed and often becomes the limiting factor determining the size of the samples that can be studied. Here, various computational schemes are utilized where the path from each reconstructed voxel to the fluorescence detector is computed as a part of the reconstruction (Schroer, 2001 ▸; Brückner, 2021 ▸). Unlike for scattering-based techniques, in the fluorescence case typically only a single outgoing ray direction has to be considered. However, a large number of different photon energies have to be corrected for independently as the absorption density varies strongly with the photon energy close to emission lines.

In this paper, we perform an in-depth investigation of the severity of the anisotropic absorption effect and test a computational approach to correct for this effect, which is then applied to experimental data.

## 2. Method

The sample in TT is described by a function characterizing its scattering, *f*(**q**, **r**), related to its microscopic structure at macroscopic position **r** and an absorption density μ(**r**). Both functions have finite support in **r** in some volume *V*. We will work in a sample-fixed coordinate system where the sample functions are fixed but the wavevectors vary as the sample is rotated.

In the experiment, a narrow pencil beam of monochromatic X-rays is incident on the sample in a direction parallel to **k**_0_ and the center of the beam passes though the point **r**_0_. The scattered light is observed far from the sample in a direction parallel to **k**_*h*_.

**Figure 1:**
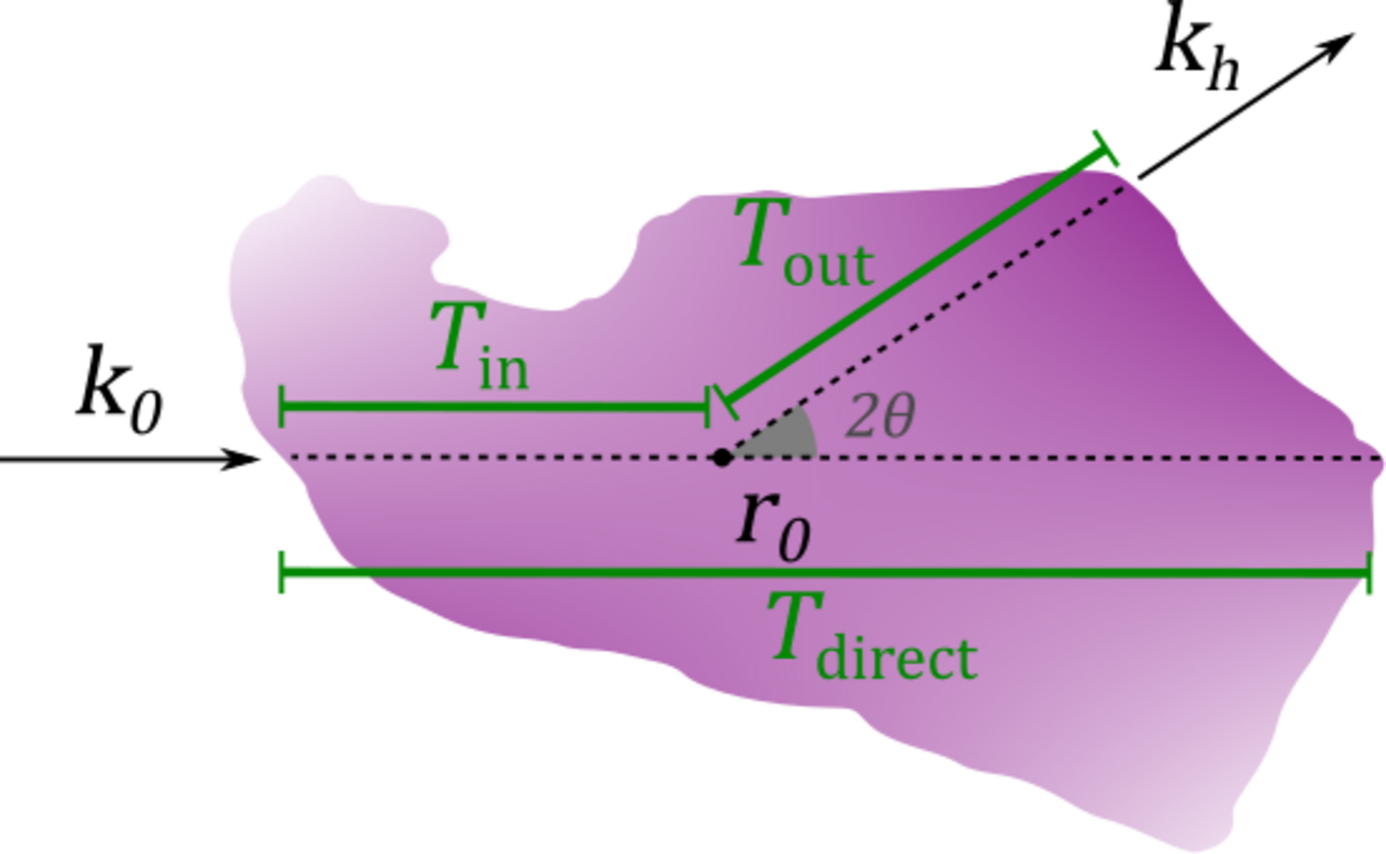
2D slice of an absorbing object. The scattered ray at point **r**_0_ in the direction **k**_0_ sees the transmission *T*_in_ × *T*_out_ given by integrals over the dotted lines.

The contribution to the scattered intensity originating from the point **r**_0_ is given by the expression
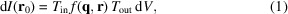
where *T*_in_ is the transmission of the incoming ray from the source outside the sample until the internal point **r**_0_, *T*_out_ is the transmission of the scattered ray from **r**_0_ to the detector pixel and **q** = **k**_*h*_ − **k**_0_ is the scattering vector. The quantities are sketched in Fig. 1 ▸. The scattered X-rays see the total absorption *T*_in_ × *T*_out_. The small-angle approximation is to normalize the scattering by the transmission of the direct beam, *T*_direct_. The largest difference between the transmission of the direct and scattered beams happens at the side where the beam enters the sample, while the two are identical where the beam exits the sample. The difference can become very large for glancing rays, where the scattered beam immediately exits the sample while the transmitted beam travels through it, or vice versa.

Both transmission coefficients can be expressed as line integrals through the absorption density. We define the partial-absorbance function, 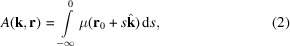
where 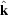 = **k**/|**k**| is the normalized wavevector. We further define the partial transmission as
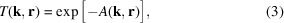
which describes the transmission coefficient of a beam traveling from outside the sample until the point **r** along the direction of **k**. We see that *T*_in_ = *T*(**k**_0_, **r**_0_) and *T*_out_ = *T*(−**k**_*h*_, **r**_0_). In Appendix *A*[App appa] we specify an algorithm to compute these quantities from a voxelized representation of μ(**r**).

The experimentally measured intensity can now be written as an integral over all points along the direct-beam path, 
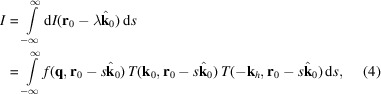
where **r**_0_ is an arbitrary point on the direct-beam path. Notably, the second line of equation (4) cannot be factored into the usual CT line integral over *f* and an absorption correction factor independent of *f* since the transmission is a function of the position along the line. The absorption can therefore not be compensated by a single multiplicative correction factor on the measured intensity.

### 2.1. Absorption correction in tensor tomography

Using the formalism of Nielsen *et al.* (2025 ▸), the forward model of TT can be written 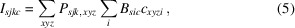
where *I*_*sjkc*_ is the (un-corrected) scattered intensity measured at a given orientation of the sample labeled by *s* and position *j*, *k*. The intensity is re-grouped into azimuthal segments labeled by *c*. *P*_*sjk*,*xyz*_ is a discretization of the usual line integral of CT, where *x*, *y* and *z* refer to the voxels of the reconstruction. Furthermore, *c*_*xyzi*_ are the unknown expansion coefficients of the scattering function *f*(**r**, **q**), where *i* labels the basis functions used in this expansion and *B*_*sic*_ maps the basis functions to the detector channels.

The most straightforward approach to reconstruction is to minimize the least-squares cost function, 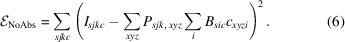
Such a reconstruction contains significant artefacts when the sample absorbs strongly, as will be shown; conventionally this is corrected for by normalizing by the transmission of the direct beam, which we refer to as the small-angle approximation, leading to the cost function
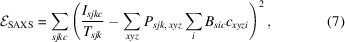
where *T*_*sjk*_ is the absorption of the direct beam, which needs to be known prior to the reconstruction.

The choice to normalize the measured intensity by the transmission is not the only way that the absorption can be included in the model. Equally well, the transmission can be included as a multiplicative factor on the second term giving a different cost function, which can be expressed as a weighted version of the same least-squares problem with weight factors 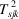. We have observed that such a cost function suffers from slow convergence on samples with high absorption when using first-order gradient based optimizers and for that reason the normalization approach is preferred.

We further introduce a new cost function that includes the anisotropic absorption effects:
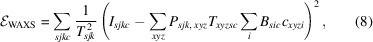
where *T*_*xyzsc*_ is the transmission factor that depends both on the position of the scattering element (*xyz*) and on the direction of the incident (*s*) and scattered (*sc*) beam. The weighting factor of 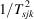 is included to make 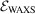 comparable with 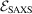 for small scattering angles [where *T*_*xyzsc*_ ≈ *T*_*sjk*_ for all voxels (*xyz*) along the direct beam (*sjk*)] and to avoid the slow convergence issue mentioned above.

The linear least-squares optimization problems are solved by gradient descent with a momentum term run to a fixed high number of iterations with the appropriate step size determined by power iterations. The matrix elements *B*_*sic*_ and the X-ray transform are computed using the open-source software package *Mumott* (Nielsen *et al.*, 2025 ▸).

## 3. Simulation study

To first investigate the effect of anisotropic absorption on WASTT, we simulate a series of WASTT datasets with varying degrees of absorption and at different scattering angles.

The simulated sample is a hollow sphere with outer diameter *D* and inner diameter *D*/2. Inside the sample, the scattering function of the sphere is isotropic and constant, so any anisotropy in the simulated diffraction data is purely a result of the absorption. The data are simulated using the same forward model as the reconstructions but are simulated on a three-times up-sampled voxel grid and raster scan to avoid getting an exact match of simulated and reconstructed data.

The down-sampled voxel grid is 41 × 41 × 41 voxels and uses 64 azimuthal bins on the detector and 332 projections covering the full range of possible projection directions. This is unusually good sampling, compared with most experiments, and is chosen to avoid missing-wedge artefacts (Nielsen *et al.*, 2024 ▸) that would otherwise complicate the interpretation of the reconstructions. When using a more realistic sampling, where transmission direction near the sample holder cannot be probed, the reconstruction artefacts owing to the missing-wedge problem far exceed the artefacts from the anisotropic absorption effect for small angles and low absorption. Examples of the simulation run with more realistic sampling are given in Appendix *B*[App appb].

**Figure 2:**
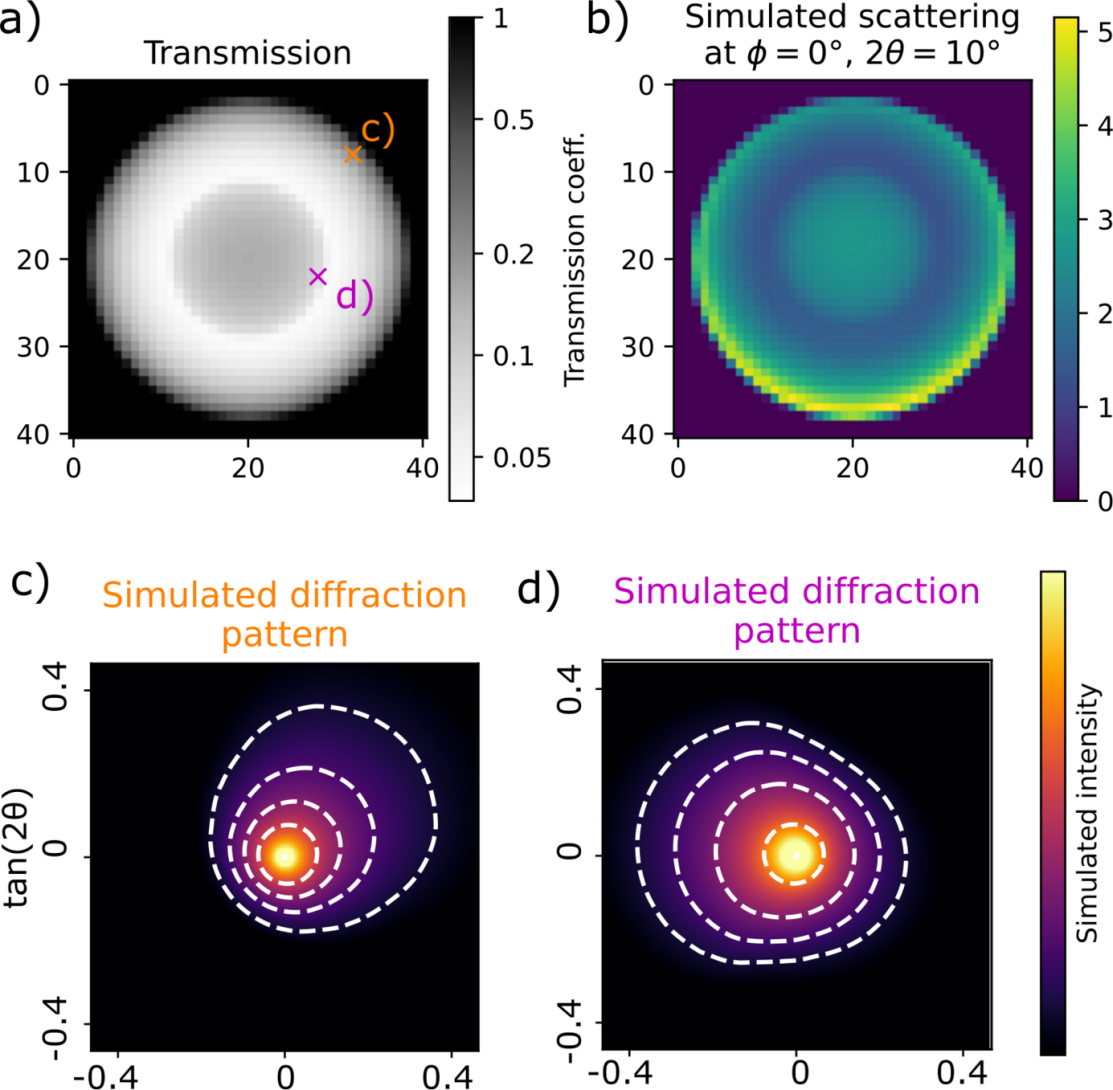
Simulated scattering from a hollow sphere displaying isotropic scattering and *D*μ = 4. (*a*) The simulated transmission of the direct beam. (*b*) Simulated intensity at a fixed azimuthal angle (corresponding to the downward direction in the figures) and a scattering angle of 2θ = 10°. (*c*, *d*) Simulated diffraction patterns corresponding to the two points marked in (*a*). Contours are overlaid with white lines highlighting the anisotropy at large scattering angles. The edge of the simulated detector corresponds to a scattering angle of 2θ = 25°.

Two parameters of the simulation sample are varied: the scattering angle 2θ and the absorption density, parameterized by the dimensionless quantity *D*μ, which is the product of the outer sphere diameter and the absorption density. Fig. 2 ▸ shows examples of the simulated data. Even though the phantom sample scatters isotropically on the length scale of a single voxel, the scattering that reaches the detector has clear anisotropy due to absorption. This anisotropic absorption is most significant for glancing rays, *i.e.* when the direct beam intersects the sample nearly parallel to the surface. The rays scattered in the direction out towards the edge leave the sample and experience significantly reduced absorption compared with the rays scattered away from the edge and further into the bulk of the sample. This effect becomes more significant for larger scattering angles. This can be observed from Figs. 2 ▸(*c*) and 2 ▸(*d*) showing simulated diffraction patterns from points where the direct beam passes nearly parallel to a sample surface. The constant-intensity contours are close to circular at low scattering angles and become more eccentric as the angle increases.

**Figure 3:**
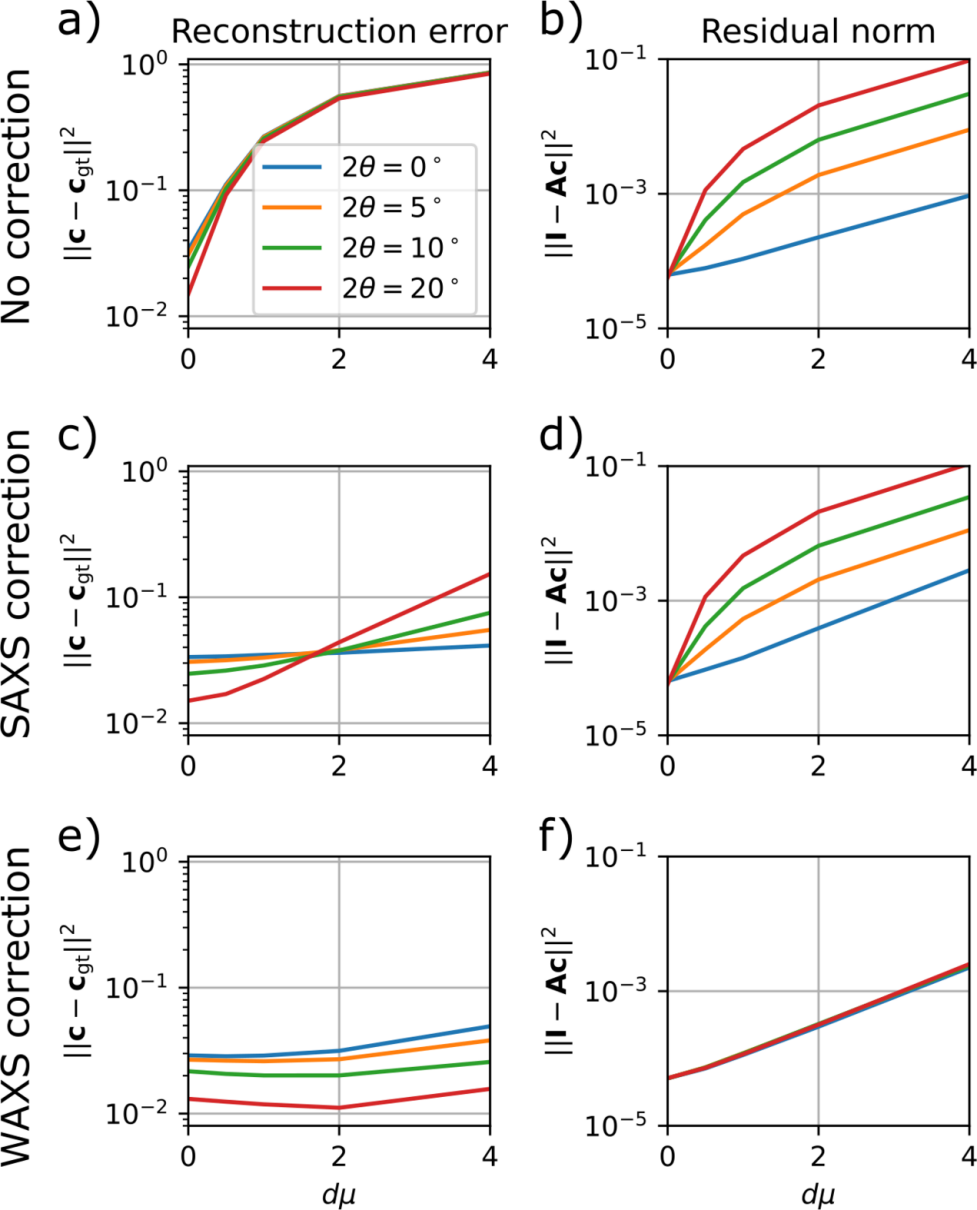
Comparison of reconstructions of the simulated datasets using the three different error functions: (*a*, *b*) no absorption correction; (*c*, *d*) small-angle approximation; and (*e*, *f*) wide-angle absorption. (*a*, *c*, *e*) The reconstruction error defined as the square sum of the optimized coefficients minus the ground truth. (*b*, *d*, *f*) The respective error functions for the converged solution.

We compare two measures of the accuracy of the reconstruction: the residual norm, which is equal to the square sum of the data minus the forward model, and the reconstruction error, equal to the square sum of the reconstructed coefficients minus the ground truth. Both are normalized such that zero solution gives a value of 1. For all reconstructions we use an unconstrained second-order spherical harmonics model for the scattering. For *D*μ = 0 (blue curves in Fig. 3 ▸), all three reconstructions are identical. The probed range of reciprocal space varies as a function of scattering angle and therefore converges to different values of the reconstruction errors in the range from 0.015 to 0.035. This error is due to the difference between the high-resolution tomogram used to simulate the data and the lower-resolution voxel grid of the reconstruction.

**Figure 4:**
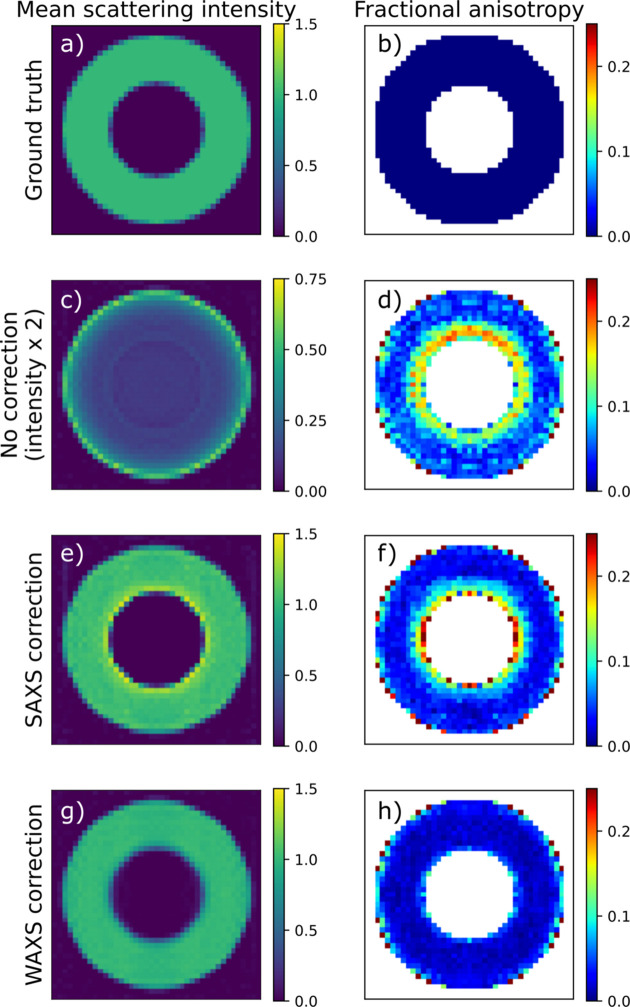
Slices of reconstructed simulated data with *D*μ = 2.0 and 2θ = 20°. (*a*, *b*) Ground truth; (*c*, *d*) no absorption correction; (*e*, *f*) SAXS correction; and (*g*, *h*) WAXS correction. (*a*, *c*, *e*, *g*) Reconstructed directionally averaged scattering density. (*b*, *d*, *f*, *h*) Fractional anisotropy. The color coding of (*c*) is different from the rest to improve visibility.

Without absorption correction, the reconstruction error rises sharply already for the moderate absorption value *D*μ = 0.5, irrespective of the scattering angle. Fig. 4 ▸(*c*) shows that the reconstruction has too low intensity and places most of the intensity at the outer boundary of the sample, as the scattering from the interior is screened. The reconstructions made using the small-angle approximation of the absorption term show significantly reduced reconstruction error [Fig. 3 ▸(*c*)], only going above 0.1 for the highest 2θ and *D*μ value. The residual norm, however [Fig. 3 ▸(*d*)], is significant already at smaller scattering angles. Fig. 4 ▸ shows slices of the reconstructed tomograms. We see that the error appears as a too high intensity near the inner boundary of the hollow-sphere phantom sample along with an increased anisotropy [Figs. 3 ▸(*e*) and 3 ▸(*f*)]. The reconstructions made using the anisotropic absorption correction have small reconstruction error for all simulations and it never rises significantly above the level of the zero-absorption reconstructions, which we attribute to discretization errors. Notably, the residual norm does not rise with increasing scattering angle [Fig. 3 ▸(*f*)].

In summary, absorption correction is critical already at quite moderate levels of absorption to avoid artefacts where the interior of the sample is reconstructed with too small density compared with the boundary, similar to beam-hardening artefacts in conventional CT. The small-angle approximation removes this artefact and produces acceptable reconstructions up to about 2θ = 10° and *D*μ = 2, with reconstruction artefacts appearing near sharp edges in the absorption density. Including the anisotropic absorption effects in the reconstruction improves the fit significantly (seen by the decreased residual norm) and alleviates some reconstruction artefacts at high absorption and scattering angles.

## 4. Experimental study

In the simulation, it appears that a voxel-by-voxel anisotropic absorption correction can improve the reconstructions. In this section, we test the applicability to experimental data.

**Figure 5:**
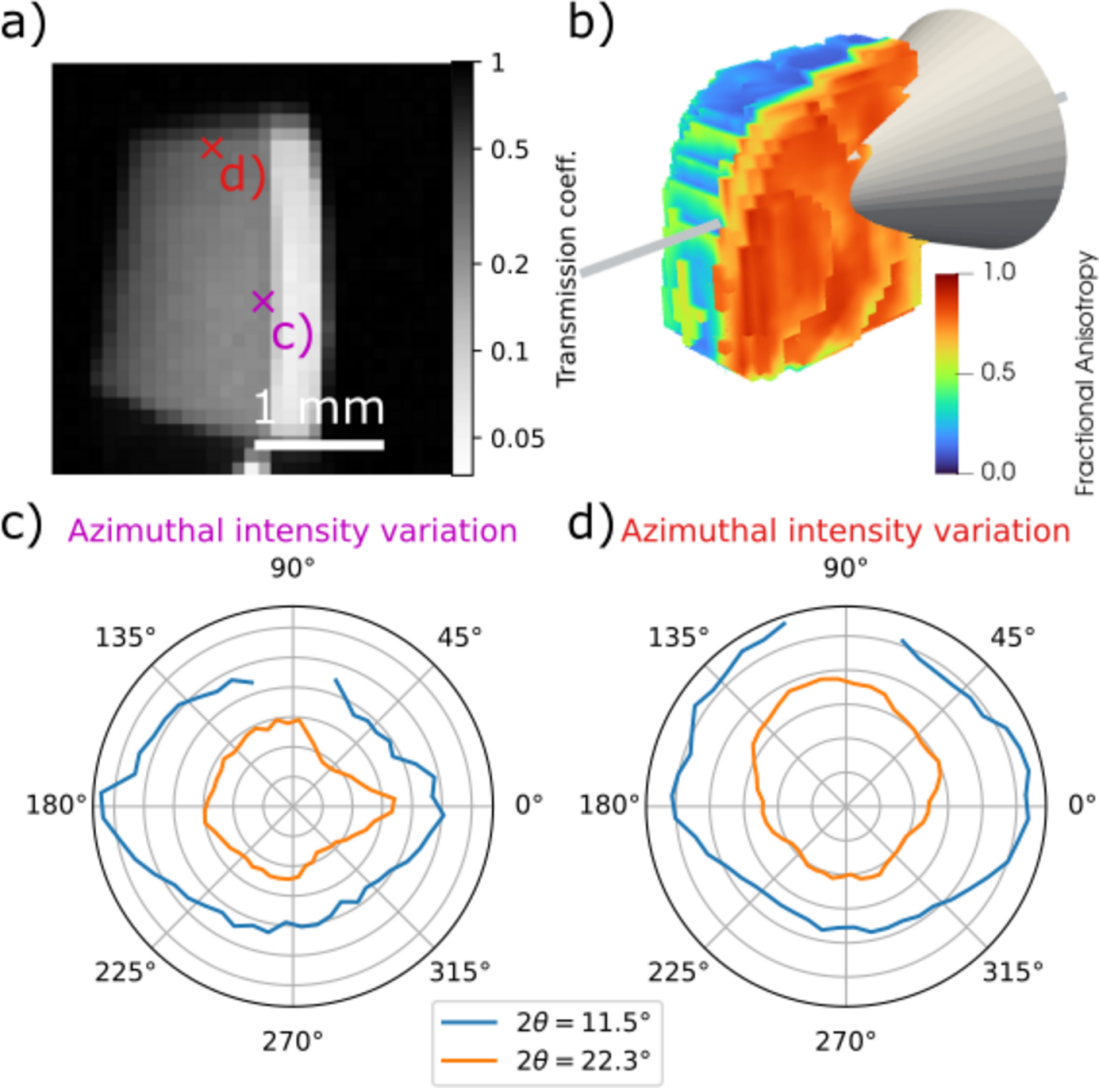
(*a*) Measured transmission coefficient of the direct beam in the orientation looking straight down the enamel layer. (*b*) A 3D rendering of the sample geometry and a scattering cone with an opening angle of 2θ = 20°. The model of the sample is colored according to the reconstructed fractional anisotropy, which highlights the difference between the highly aligned enamel (red) and the more isotropic dentine (blue/green). Panels (*c*) and (*d*) show the azimuthal variation of the scattered intensity at the points marked by crosses in (*a*) for the 002 and 004 Bragg peaks, respectively, where the distance from the center is proportional to the square root of the measured intensity. Missing data points are due to overlap of the Bragg peaks with gaps in the X-ray detector.

The data used for the test are from an incisor of a Eurasian beaver (*Castor fiber*). A small cylinder of diameter 1 mm and length 1.5 mm was prepared containing the full thickness of the enamel layer and some amount of dentine to give a geometrically non-trivial sample with significant absorption. When transmitting through the full length of the enamel layer edge-on, the transmission is around 0.05, while, in the perpendicular direction, the transmission is around 0.2 [see Fig. 5 ▸(*a*)].

The experiment was performed at the cSAXS beamline of the Swiss Light Source (SLS) using a photon energy of 18 keV. The beam was focused to a spot of ∼50 µm × 50 µm. Then, 225 projections were measured uniformly spaced over a range of projection directions up to a maximum tilt angle of 45°. The images were integrated in 64 azimuthal bins and two radial bins covering the full width of the Bragg peaks. The transmission was measured on the same Pilatus 2M detector used to measure the diffraction patterns behind a semi-transparent beamstop consisting of an 8 mm-thick disk of single-crystal silicon aligned to avoid any Bragg reflections.

The reconstructed diffraction features are the apatite 002 and 004 Bragg peaks, which at the photon energy of 18 keV appear at scattering angles of 11.5° and 22.3°, respectively. Because the two reflections are co-linear, they are expected to display the same directional distribution. The sample consists of two clearly distinguishable materials, both containing hydroxyapatite: the enamel, which forms a narrow layer at one edge of the tooth and has strongly textured and narrow Bragg peaks, and the dentine, which forms the bulk of the sample and displays much weaker texture and broader peaks.

Fig. 5 ▸ shows examples of the measured data. The effects of anisotropic absorption are overlaid with the microscopic anisotropy of the sample, which makes interpretation less clear than for the simulated data. Some trends are still observable, mainly the stronger scattering in the ‘up’ direction compared with the ‘down’ direction at a point close to the top edge of the sample [Fig. 5 ▸(*d*)], and the weak scattering observed in directions that pass through the enamel layer at a shallow angle from a point close to the dentine–enamel interface [Figs. 5 ▸(*b*) and 5 ▸(*c*)]. In both cases, the effect is more noticeable for the larger scattering angles.

### 4.1. Reconstructions

The beaver tooth sample is reconstructed by minimizing the error functions given by equations (7) and (8). Both reconstructions were found to contain significant over-fitting artefacts and therefore a Tikhonov-regularization term was included in both. A good value of the regularization parameter was found by performing a large sweep of regularization parameters with the SAXS correction, and this value of the regularization parameter (λ = 5 × 10^1^) was used for both reconstructions.

The reconstruction using the wide-angle correction needs a tomogram of the absorption density. This is created from transmission data, which were measured using a semitransparent beamstop during the WASTT experiment, and reconstructed using the SIRT algorithm.

**Figure 6:**
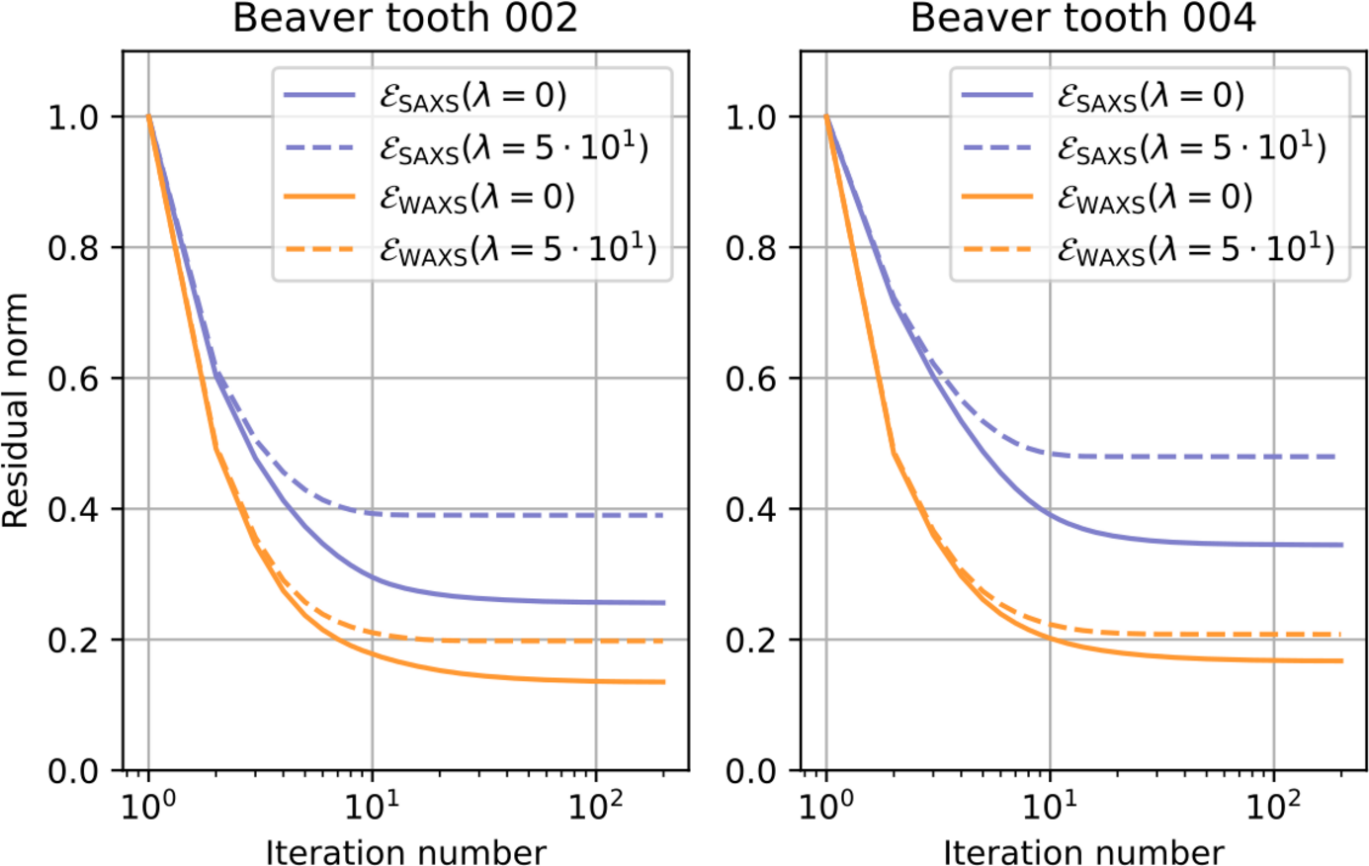
Convergence plots of the WASTT reconstructions of the hydroxyapatite {002} (left) and {004} (right) pole figures.

The trends seen in the simulations are also observed in the experimental reconstruction: the anisotropic absorption correction leads to a smaller converged value of the cost function than the small-angle approximation and the difference is more pronounced at larger scattering angles (Fig. 6 ▸).

**Figure 7:**
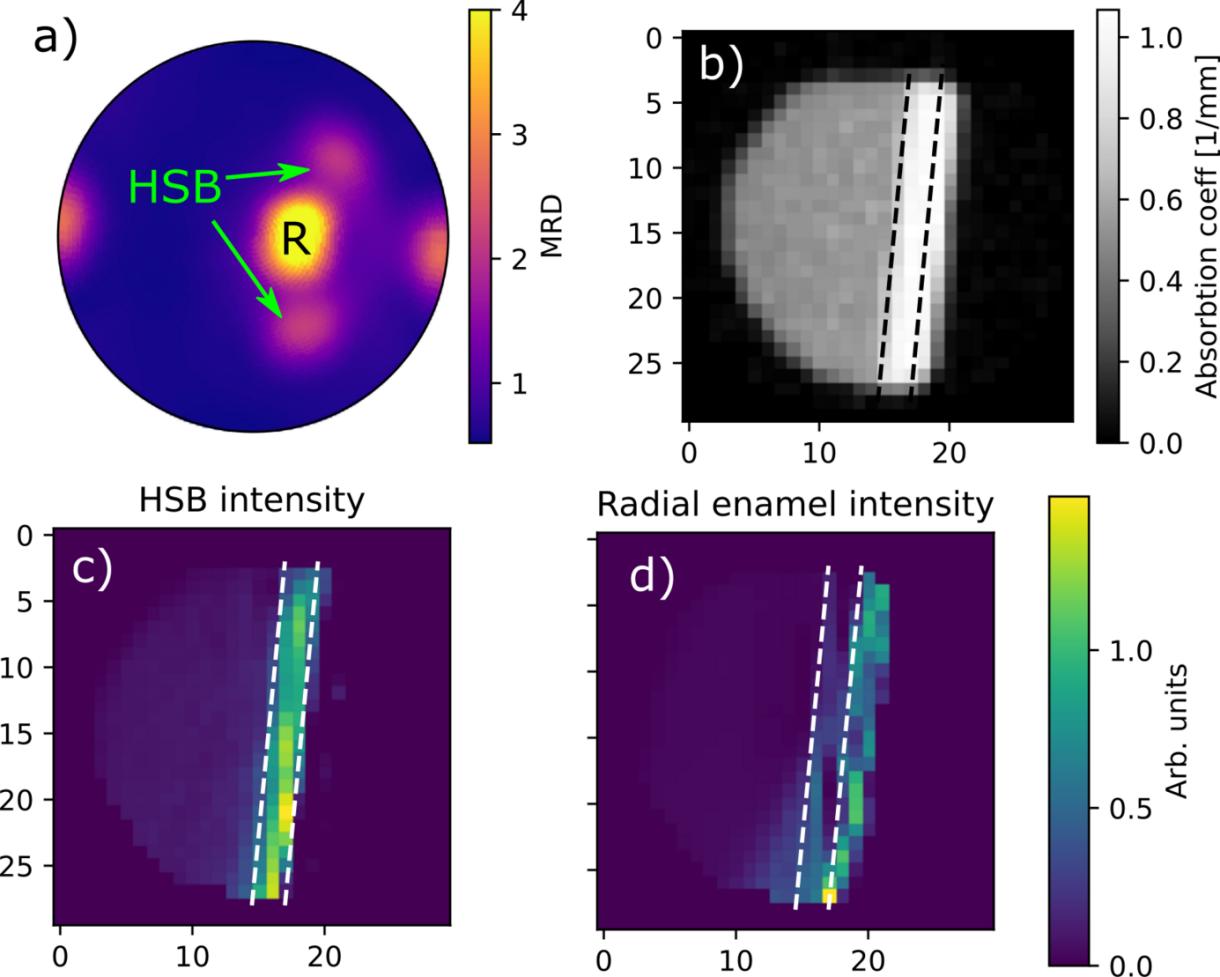
Overview of the features of the reconstructed sample. (*a*) Pole figure of the {002} peak averaged over the entire sample volume in units of multiples of random distribution (MRD). (*b*) 2D slice of the reconstructed absorption density. (*c*) Spatial distribution of the texture component related to the HSBs. (*d*) Spatial distribution of the texture component related to the radial enamel. The dashed lines are visual guides to help distinguish the radial enamel and HSB regions and appear at the same location in all three figures.

Fig. 7 ▸(*a*) shows the pole figure of the 002 hydroxyapatite peaks recomputed from the reconstruction and averaged over the entire volume. There are several distinct diffraction features. The strongest texture component marked with R in the figure has the *c* axis aligned with the labial–lingual direction of the tooth and is due to the radial enamel that forms the outermost layer of the tooth on the labial side. Directly above and below the radial peak on the pole figure are two strong features related to the two families of Hunter–Schreger bands (HSBs) that make up the remaining part of the enamel between the radial enamel and the enamel–dentine junction. While the radial enamel and HSB regions cannot be distinguished in the absorption tomogram [Fig. 7 ▸(*b*)], they are clearly distinguishable in the diffraction direction and are spatially resolved in the WASTT reconstructions as highlighted by the dashed lines in Fig. 7 ▸. The bulk of the sample consists of dentine and displays largely isotropic scattering (fractional anisotropy of around 0.15 compared with 0.8 for the radial enamel). The remaining sharp feature in the pole figure at the left- and right-hand sides appears to be spread out in the dentine as well; however, it appears at a position where the missing-wedge artefacts would lead it to be spread out even if it was localized to a narrow plane like the enamel features, meaning the location of this scattering feature cannot be determined with the applied geometry (Nielsen *et al.*, 2024 ▸).

**Figure 8:**
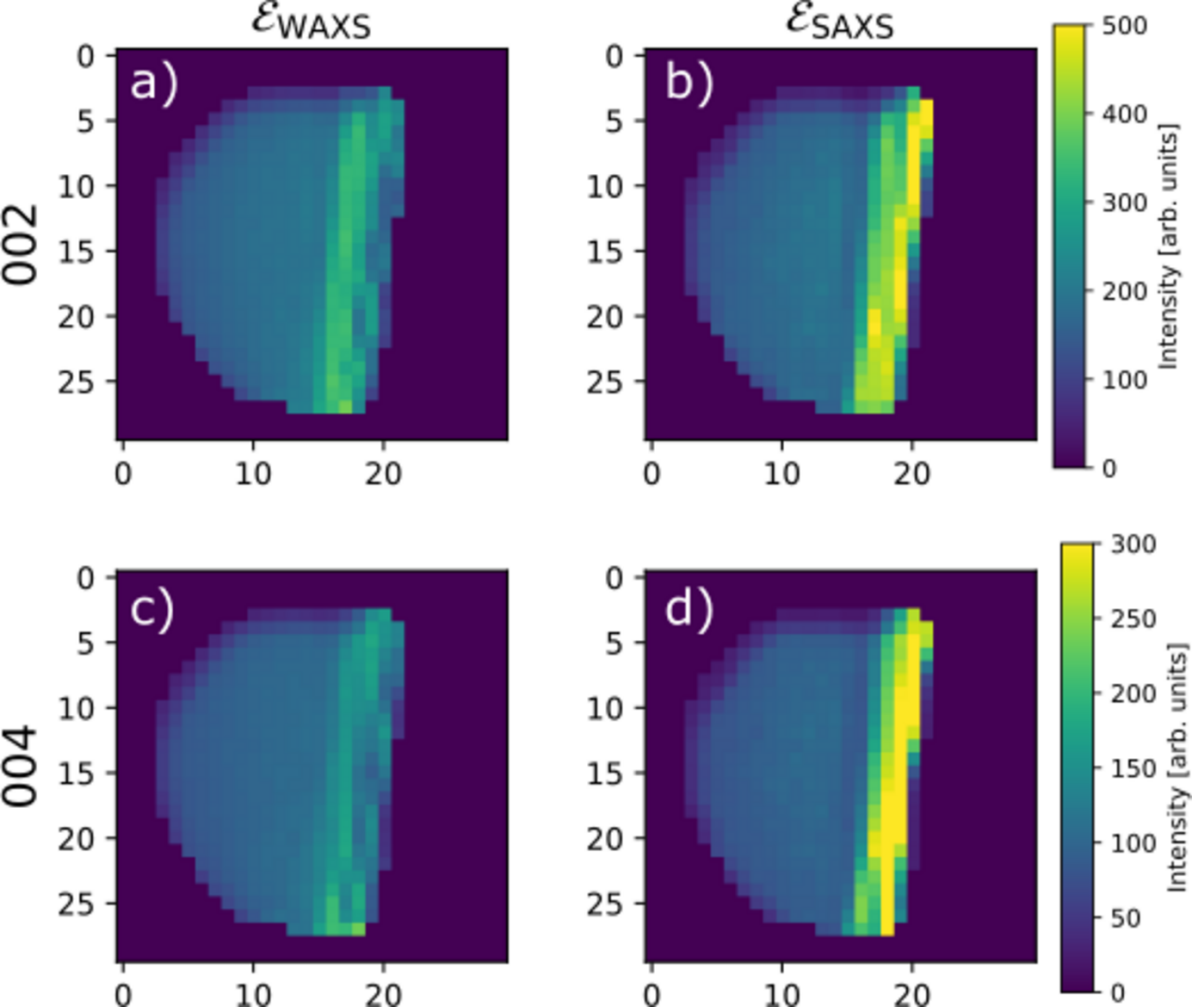
Slices of the reconstructed mean intensity of the beaver tooth sample of the (*a*, *b*) {002} pole figure and the (*c*, *d*) {004} pole figure using the (*a*, *c*) anisotropic absorption correction and the (*b*, *d*) small-angle approximation.

We perform reconstructions using the wide-angle absorption correction and the small-angle approximation, and the resulting tomograms are qualitatively very similar. Fig. 8 ▸ shows slices of the reconstructed mean intensity of the regularized reconstructions using the two different approaches and Bragg peaks. The main systematic difference is that the SAXS correction reconstructs higher intensity in the enamel part compared with the reconstruction using the WAXS correction. If we assume that the absorption is primarily due to apatite at this X-ray energy, we expect the profile of the mean intensity to follow the profile of the absorption density. The absorption density displays two distinct regions, the dentine and the enamel [Fig. 7 ▸(*b*)], where the density in the enamel region is on average 1.9 times higher than in the dentine region. For the TT reconstructions, this fraction is 2.2 (apatite 002) and 2.5 (apatite 004) using the small-angle approximation, and 1.7 (apatite 002) and 1.6 (apatite 004) for the reconstruction with the wide-angle absorption correction. It thus appears that the small-angle correction is overestimating the absorption. This makes sense as the enamel diffraction features are only observed at an orientation where the direct beam is parallel to the enamel layer, leading to a higher absorption of the transmitted ray than the scattered rays.

Furthermore, a small area of lower mean intensity is present between the enamel–dentin junction in the SAXS correction reconstructions, which is not seen when using the wide-angle absorption correction. This is consistent with our expectations of artefacts appearing near sharp changes in the absorption density. While these observations suggest that the wide-angle absorption correction improves the reconstruction, the lack of ground truth prevents any quantitative comparison.

In both the simulation and the experimental study, we observe that the inclusion of anisotropic absorption effects in the forward model leads to a significantly improved fit to the experimental data. For scattering angles in the range 10–20° and transmission of the order of 5–10%, we observe a factor of two reduction in the residual norm of the converged solutions. Since the different models use the same degrees of freedom to describe the sample’s scattering properties, the improvement of the fit to experimental data indicates that the wide-angle absorption model more accurately describes the experiment.

In the simulation study, we see that this improvement of the fit comes with a small improvement in the converged solution itself. With either transmission higher than 10% or scattering angles smaller than 10°, the simulations still show a reduction of the residual norm but with no noticeable improvement of the reconstruction. In the experimental study where we selected a particularly difficult sample with a highly absorbing sharp edge on one side, the observed changes in the reconstruction when using the wide-angle absorption correction are consistent with removing of artefacts, which would be expected from such geometry.

## 5. Experimental considerations

The need to correct for absorption necessitates that the transmission of the direct X-ray beam be either measured during the experiments or modeled by some other approach. To measure the transmission during the TT experiment, three general approaches are commonly employed: (1) the strength of the direct beam is measured on the same 2D detector that measures the diffraction patterns behind a semi-transparent beamstop; (2) an X-ray sensitive diode is placed directly on the beamstop; and (3) a beamstop that consists of or contains a fluorescent material is used, and the fluorescence is measured using a fluorescence detector or a photo-diode (Birkedal *et al.*, 2024 ▸).

Advantages and disadvantages can be listed for all approaches: a semi-transparent beamstop needs to be matched to the photon energy used to avoid oversaturating the 2D detector and, if the X-ray beam contains even a small fraction of higher harmonics, the readout behind the beamstop will be polluted.

X-ray sensitive diodes are liable to oversaturate and the signal tends to drift over long timescales. Furthermore, they are difficult to make small enough to be compatible with small-angle scattering experiments. A fluorescent beamstop is easier to make small, but some care needs to be taken that the signal is strong enough that counting statistics are still good when a highly absorbing sample is being investigated.

Another issue arises when the sample refracts the direct X-ray beam, causing ultra-small-angle scattering. If the active area of the transmission measurement is not large enough to collect the full extent of the perturbed transmitted beam, this effect can lead to a wrong transmission measurement. Thus, especially for small-angle scattering, a compromise must be made between a beamstop that is large enough to collect most of the transmitted intensity and not so large that it blocks the scattering signal.

Another option is to perform an additional absorption-CT measurement of the sample (Grünewald *et al.*, 2023 ▸) or to simulate such data from sample knowledge (Silva Barreto *et al.*, 2024 ▸), *e.g.* when the density is uniform. This tomogram can afterwards be used to compute the transmission of the direct beam in the TT experiment. This adds an extra alignment step for the reconstruction with potential to introduce errors. Ideally, the photon energy used for the absorption tomogram should match the energy of the TT experiment. This is especially important when a geometrically complicated sample containing multiple materials is used.

If the transmission is measured during the TT experiment, it can directly be normalized out of the scattering data if using the small-angle approach. If the transmission is measured in a separate experiment, it has to be scaled to the same resolution and aligned with the scattering measurement both in orientation and position, leading to further complications in the reconstruction. For the wide-angle absorption approach presented here, a tomogram needs to be reconstructed from the measured transmission. If the same projection model is used for the absorption reconstruction and for the TT reconstruction, no extra care needs to be given for the alignment of the two. However, the computation of a separate transmission correction for each voxel and scattering direction during the reconstruction introduces significant computational costs on the reconstruction. For the beaver tooth sample investigated in this paper, the increase in computation time is roughly a factor of 15.

The most straightforward strategy to mitigate the effect of anisotropic absorption is to use an X-ray beam with a higher photon energy. This has the double effect of reducing the absorption coefficient and making the scattering angles smaller, both of which will reduce the anisotropic absorption effect. While this may be the best strategy in some cases, concerns relating to the beam inducing damage in the sample during measurement, signal-to-noise ratio and limitations of the instrument also have to be considered.

## 6. Conclusions

The simulation study presented in this paper shows that the conventional small-angle approximation for transmission correction remains useful in WASTT up to quite large scattering angles and absorption levels, and we estimate that it can be used safely when the transmission is above 0.5 and the scattering angles below 10°, which is the case for most published WASTT studies. The simulations further show that, if absorption is not corrected for at all, it leads to reconstructions with too little intensity in the interior of the sample. It is therefore critical to correct for absorption when operating at high-absorption conditions, even when the scattering angles are small.

We have demonstrated that a modified algorithm using the error function defined in equation (8) that includes anisotropic absorption effects can be used in settings with high absorption and large scattering angles to achieve a better fit to the measured data, and potentially improved reconstructions, at the expense of significantly increased computational cost. This amounts to an increase of about a factor of 15 for computation time for the sample at hand and a more complicated reconstruction procedure. More details of the calculation of correction factors are given in Appendix *A*[App appa].

## Figures and Tables

**Figure 9:**
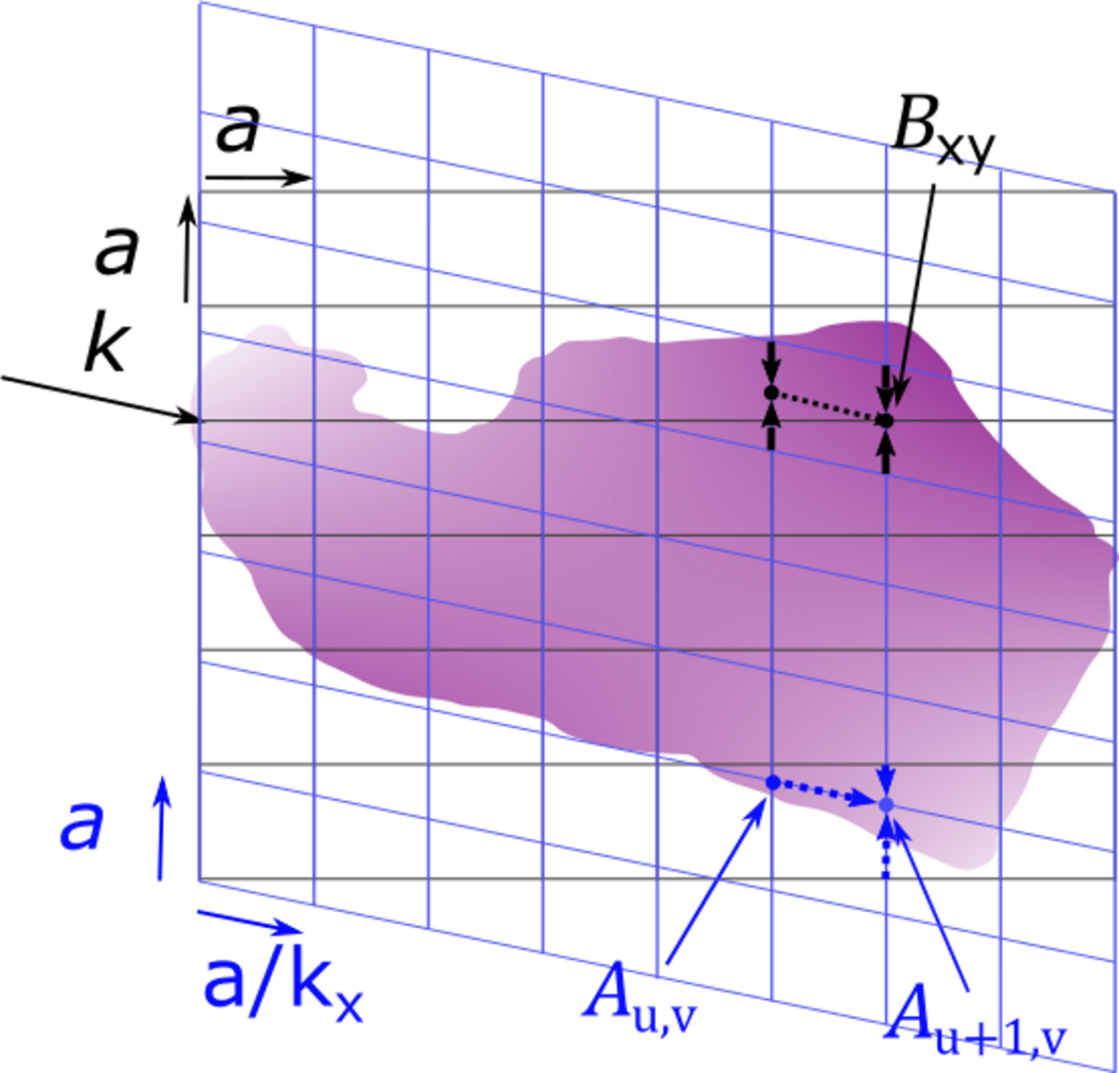
Illustration of the two-step interpolation scheme. First, a new grid is chosen for a given direction, **k**, and the partial absorption *A*_*u*,*v*_ is calculated at every grid point using bi-linear interpolation and accumulated along **k**. In the second step, the partial absorbance seen by a given voxel, *B*_*xy*_, is backinterpolated from the new grid by trilinear interpolation to a point one half-slice behind the voxel to avoid double counting the voxel of interest.

**Figure 10:**
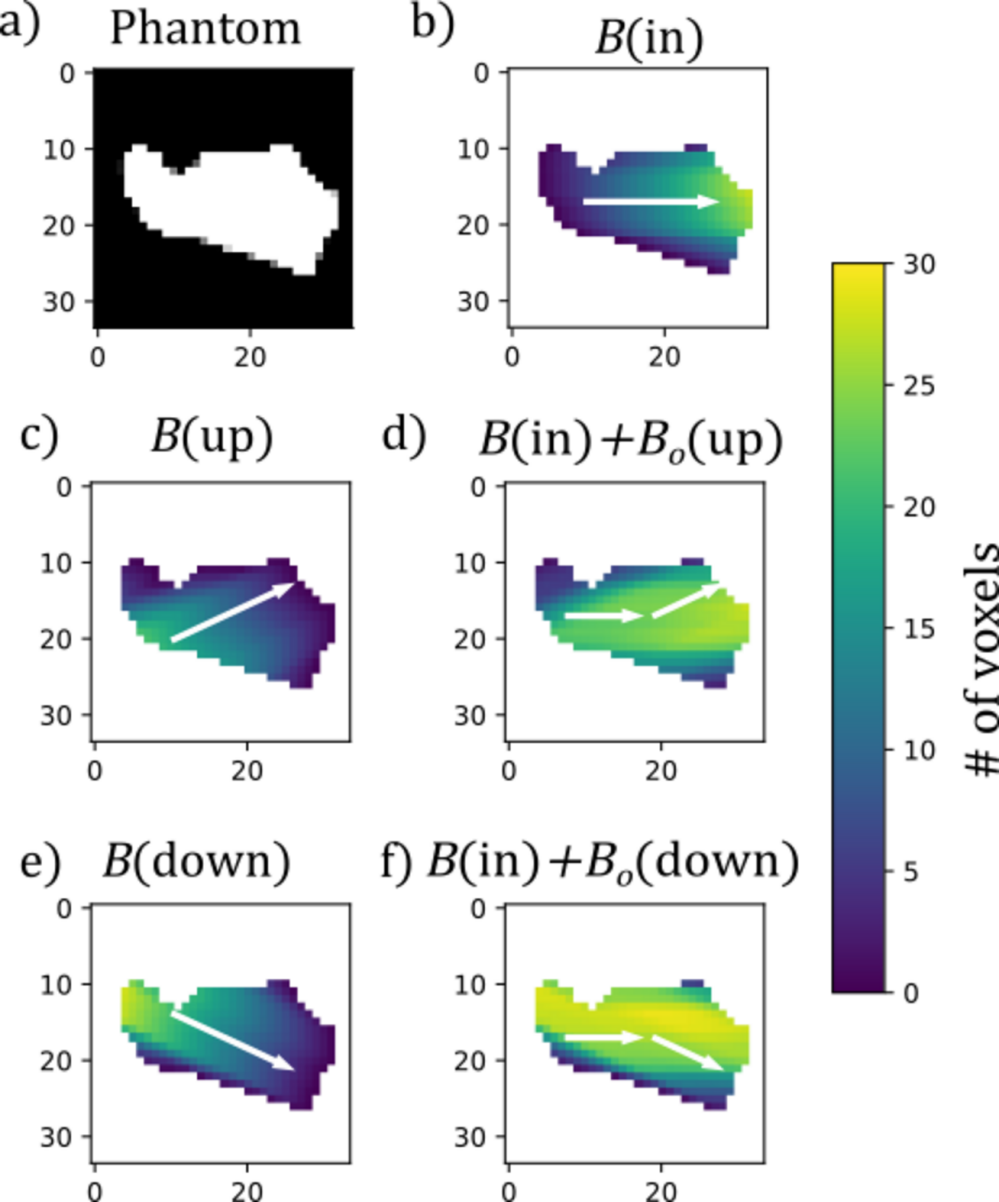
Example 2D calculation showing the difference between scattering in the directions ‘up’ and ‘down’. (*a*) The phantom used. It has constant absorption density inside the white region and zero outside. (*b*, *c*, *e*) Calculated absorbances in three different directions. (*d*, *f*) Total absorbance seen by each voxel in the two different scattering directions.

**Figure 11:**
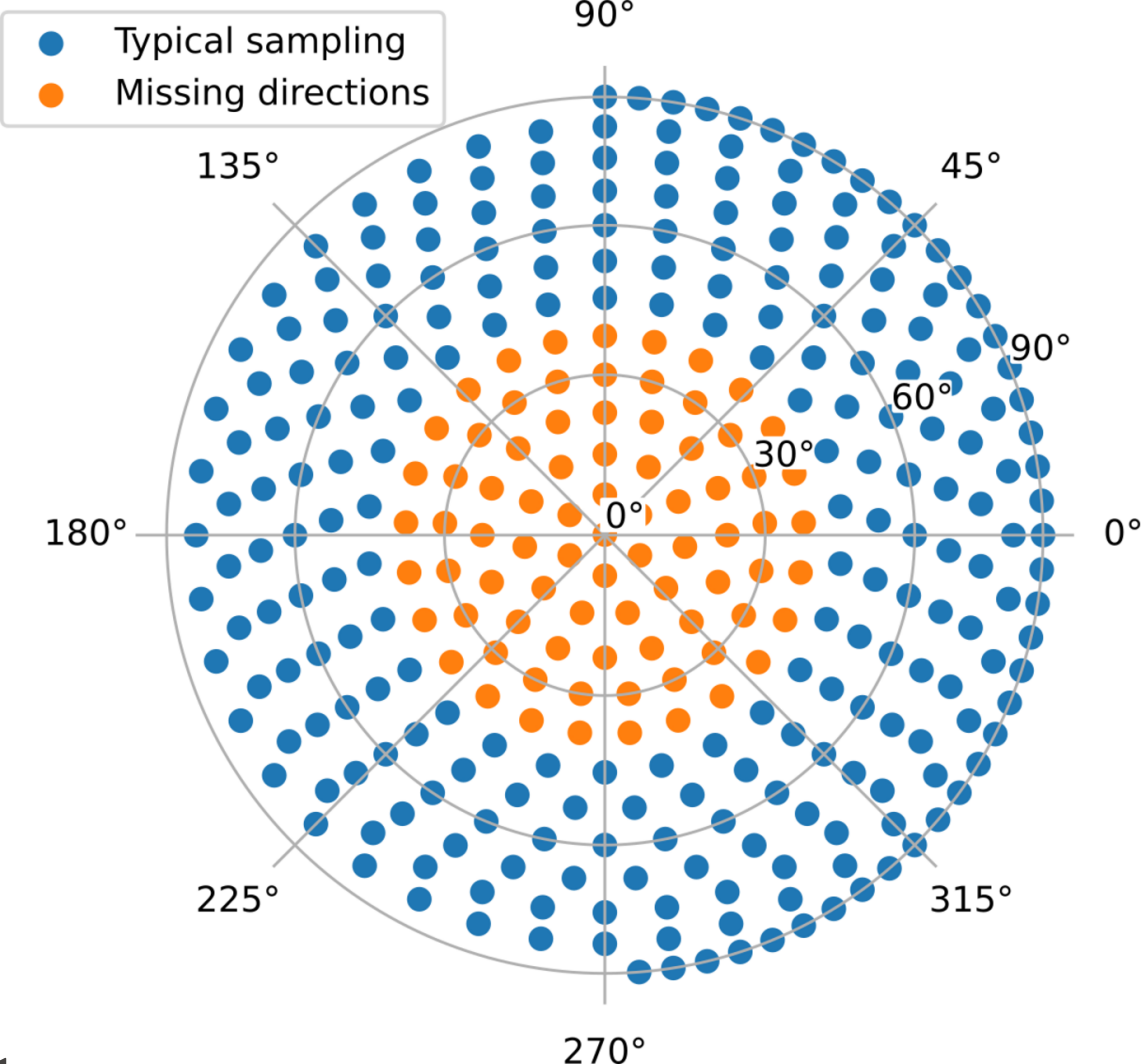
Projection directions used in the simulation study plotted in equal-area polar projection. The orange points are excluded in the reconstructions shown in Fig. 12. The half circle around the equator is sampled more finely than the rest of the unit sphere, to aid in data alignment. The apparent anisotropic sampling density at the outer part of the figure is an artefact of the distortions in the equal-area projection.

**Figure 12:**
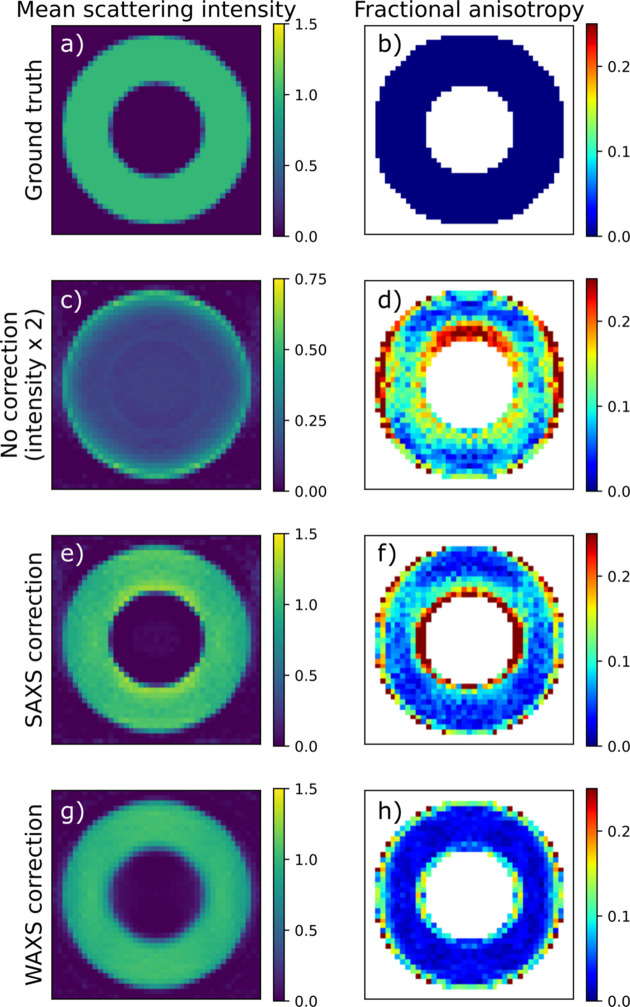
Effect of missing projection angles on the simulated reconstructions. Slices of reconstructed simulated data with *D*μ = 2.0 and 2θ = 20°. (*a*, *b*) Ground truth. (*c*, *d*) No absorption correction. (*e*, *f*) SAXS correction. (*g*, *h*) WAXS correction. (*a*, *c*, *e*, *g*) Reconstructed directionally averaged scattering density. (*b*, *d*, *f*, *h*) Fractional anisotropy. The color coding of (*c*) is different from the rest to improve visibility.

## Data Availability

The simulation code and reduced experimental data are available for download at https://zenodo.org/records/15826720.

## Appendix A Ray-tracing algorithm

In this section, we present an algorithm to calculate the partial transmission defined by equation (3) from a voxel representation of the absorption function. The voxel grid is a cubic grid,

where *x* = 0, 1,…, *N*_*x*_ − 1; *y* = 0, 1,…, *N*_*y*_ − 1; *z* = 0, 1,…, *N*_*z*_ − 1 and *a* is the constant side length of the voxels.

We label the partial-transmission coefficients *T*_in_ =  and *T*_out_ = , where  and  are the wavevectors of the incoming and scattered ray, respectively, and depend on the indices *s* (sample rotation) and *c* (detector azimuth), where *s* = 0, 1,…, *N*_*s*_ − 1 and *c* = 0, 1,…, *N*_*c*_.

The calculation of *T*_in_ and *T*_out_ is similar to the calculation of the X-ray transform in normal 3D CT and we can use the same interpolation schemes that are commonly used to define *P*_*sjk*,*xyz*_. For this study, we use the slice-interpolated bi-linear scheme (Xu & Mueller, 2006 ▸).

There are, however, a few noticeable differences between calculating the X-ray transform and partial transmission. In the former case, we are interested in the integral over a line through the entire sample volume intercepting a known point corresponding to the beam position. For the latter case, we are interested in the integral over a line segment that ends on a point inside the sample, which corresponds to the center of a given voxel.

Furthermore, the number of distinct lines is much larger for *T* than for *P*. In *P* we need one line integral for each sample position in the raster scan, the number of which is *N*_*j*_*N*_*k*_. For *T* we need a line integral for every voxel in the sample, the number of which is *N*_*x*_*N*_*y*_*N*_*z*_. In a typical TT experiment, this number is a factor of 50–100 times larger than the previous. So, to avoid a large increase of computation times, we implement a double-interpolation scheme where the absorption seen by all voxels for a single **k** is computed simultaneously. This is achieved by first computing the partial absorption at a synthetic array of points covering the sample and then backinterpolating the absorption from this array to the voxel grid. Fig. 9 ▸ illustrates this approach.

As with the slice-by-slice algorithms, the voxel direction (*xyz*) corresponding to the largest absolute vector component of the ray direction (**k**) is singled out to be the fast direction. Six different branches of the algorithm then exist depending on the fast direction and the sign of the corresponding vector component. Here we only write the algorithms for the case +*x*.

The synthetic array is given by the three (non-orthogonal) directions, **k**,  and , such that

The value of the accumulated absorption at the plane *u* = 0 is zero and all other points are found by the rule

where ρ(**p**_*u*,*v*,*w*_) is found by bi-linear interpolation in *y* and *z* from the given voxel map padded with zeros.

After this accumulation, the partial absorption at a given voxel is given by

where again *A*(**r**) is found by bi-linear interpolation. A point one half-slice behind the voxel is used to avoid double counting the voxel itself when the absorption of both the incoming and outgoing ray is included.

Fig. 10 ▸ shows examples of the computed absorbances using a phantom sample and the suggested algorithm. Figs. 10 ▸(*b*), 10 ▸(*c*) and 10 ▸(*e*) show the partial absorbances from the incident beam direction and two scattered beam directions. Arrows are overlaid showing the corresponding wavevector. Figs. 10 ▸(*d*) and 10 ▸(*f*) show the total absorbance scattering in either the ‘up’ or the ‘down’ directions. While this total absorbance is similar for the two cases in most of the interior points, there are large differences at points close to the left boundary of the sample, especially noticeable at the upper left corner and at the long straight edge at the bottom. In general, the anisotropic absorption most strongly affects parts of the sample close to the incident-beam side of the sample.

The total number of transmission coefficients needed is large. For the beaver tooth sample investigated in this paper, it is around 290 million, and exceeds both the number of data points (11 million) and the number of model parameters (2 million) by more than an order of magnitude. Storing the correction factors as a four-byte number would cost about 1 GB of memory, but this number scales unfavorably with the size of the voxel array and the number of projections. To avoid significantly increasing the memory cost of the reconstructions, we compute the coefficients on-the-fly.

The present implementation has not been thoroughly optimized and therefore a comparison with the existing TT software (Nielsen *et al.*, 2025 ▸) is not fair. Using the present implementation, running the wide-angle absorption correction increases the computation time for a gradient calculation for the beaver tooth sample by a factor of roughly 12, and the wide-angle absorption model has worse convergence characteristics leading to an overall increase of computation time by a factor of around 15 [12 minutes with correction and 50 s without on a Tesla V100S GPU (Nvidia Corporation, Santa Clara, California, USA)] to reach a relative change of the error function of 10^−3^. Computation times are expected to scale roughly linear with the number of voxels.

## Appendix B Missing projection directions

In the experimental study presented in this paper, as well as most published studies on WASTT, the sampling of projection directions is equally spaced over a half sphere but excludes a range of directions that are blocked due to the geometry of the diffractometer. Fig. 11 ▸ shows the sampling scheme used for the simulations. In WASTT, it is sufficient to sample a half sphere of projection directions, as the opposite directions give identical scattering patterns. This inversion symmetry is broken by the anisotropic absorption effect and opposite directions could in principle provide new information, but this effect was not investigated in the present study.

The missing directions lead to a type of artefact in the reconstructions, typically called missing-wedge artefacts (Nielsen *et al.*, 2024 ▸). Fig. 12 ▸ shows the results when using only the typical sampling (blue points in Fig. 11 ▸). These artefacts remain at low-scattering angles and absorption, obscuring the effect of absorption on the reconstructions. To avoid confusing these artefacts with effects of anisotropic absorption, the simulation was performed with full coverage of the half sphere.
